# Weight management strategies in Middle-Aged Women (MAW): Development and validation of a questionnaire based on the Oxford Food and Activity Behaviors Taxonomy (OxFAB-MAW) in a Portuguese sample

**DOI:** 10.3389/fpsyg.2022.1069775

**Published:** 2023-01-04

**Authors:** Mafalda Leitão, Jamie Hartmann-Boyce, Faustino R. Pérez-López, João Marôco, Filipa Pimenta

**Affiliations:** ^1^William James Center for Research, Ispa – Instituto Universitário, Lisbon, Portugal; ^2^Nuffield Department of Primary Care Health Sciences, University of Oxford, Oxford, United Kingdom; ^3^Department of Obstetrics and Gynecology, Faculty of Medicine, University of Zaragoza, Zaragoza, Spain

**Keywords:** middle-aged women, weight management strategies, obesity, COVID-19, Portugal, the Oxford Food and Activity Behaviors taxonomy, instrument

## Abstract

**Background:**

The Oxford Food and Activity Behaviors (OxFAB) taxonomy systematize the cognitive-behavioral strategies adopted by individuals who are attempting to manage their weight. The present study aimed to (1) develop a questionnaire based on the OxFAB taxonomy, specifically adapted for middle-aged women—the OxFAB-MAW—stage of life and sex, which present a high incidence of obesity, (2) assess the psychometric properties of this tool, and (3) evaluate the discriminative power of the OxFAB-MAW (normal weight vs. obesity).

**Methods:**

Overall, 1,367 Portuguese middle-aged women between 45 and 65 years (*M* = 52.3, *SD* = 5.15) filled in a sociodemographic, health, and menopause-related questionnaire, as well as the OxFAB-MAW.

**Results:**

Confirmatory factor analysis demonstrated an acceptable model fit (comparative fit index = 0.928, Tucker–Lewis index = 0.913, root mean square error of approximation = 0.072, and standardized root mean square residual = 0.054). Five domains with one item were grouped into other domains, and the Weight Management Aids domain was also removed. The OxFAB-MAW showed factorial, convergent, discriminant, and external validity, as well as composite reliability.

**Conclusion:**

The OxFAB-MAW questionnaire is a valid, reliable, and theory-driven tool for assessing weight management strategies in middle-aged women, being able to discriminate between clinical and non-clinical groups (normal weight vs. obesity) in several domains. This instrument can be used to gather valid and reliable data, useful in both research and clinical settings (especially focused on structuring interventions and preventive obesity programs within this specific life cycle stage).

## 1. Introduction

Obesity is a global pandemic, and its prevalence has increased considerably in recent years (Jaacks et al., 2019). It is associated with psychological problems (e.g., depression and anxiety), cognitive and emotional deficits (e.g., difficulties in recognizing some emotions and limitations in executive functioning), and increased risk for cardiovascular disease, cancer, and diabetes (Boeka and Lokken, 2008; Leitão et al., 2013; Pimenta et al., 2014; Scarpina et al., 2021). Obesity is responsible for 3.4 million deaths annually and 2–8% of European health costs [World Health Organization [WHO], 2014]. Furthermore, because of the emotional and psychological responses to the severe acute respiratory syndrome coronavirus 2 (SARS-CoV-2) pandemic and mandatory confinement, the risk of developing dysfunctional eating behaviors may increase, as well as the overweight/obesity status (Ammar et al., 2020).

Obesity incidence is more prevalent in women than in men (World Health Organization [WHO], 2014; Chooi et al., 2018; Jaacks et al., 2019). In Portugal, obesity has been considered a disease since 1997 [Direção-Geral da Saúde [DGS], 2014]. In Portuguese women, the prevalence of being overweight is 57.6% and of obesity is 24.2% (World Health Organization [WHO], 2016). Middle-aged women are more susceptible to weight gain compared with younger women, especially during peri-menopausal/post-menopausal years, when most women gain weight, on an average of 0.42 kg per year (Pimenta et al., 2014; Kapoor et al., 2017; Kozakowski et al., 2017; Reda et al., 2017; Cheng et al., 2018; Schreiber and Dautovich, 2018). Although there is an interaction of multiple factors (e.g., genetics, diseases, medication intake, and/or lifestyle changes), which partly explains the obesity/excessive weight gain, the abdominal fat increase observed in middle-aged women is also due to the decrease in endogenous estrogen production, which represents a health risk increase for these women (including developing type 2 diabetes, cardiovascular disease, and cancer; Kapoor et al., 2017; Kozakowski et al., 2017; Proietto, 2017). Moreover, there is evidence that dysfunctional eating behaviors (such as binge eating disorder or emotional eating) are present among middle-aged women, and are associated with weight gain, obesity, and depressive symptoms (Leitão et al., 2013; Schreiber and Dautovich, 2018).

Overweight or obese women are motivated to neutralize the risk of obesity and many desires to lose weight, but attempts are often unsuccessful (Santos et al., 2016; Schreiber and Dautovich, 2018; Molarius et al., 2020). Exercising and dietary methods (specifically in the Energy Compensation and Restraint domains of the OxFAB) are the most used weight control behaviors in women (Santos et al., 2016). Also, social, physical, and macro-environmental characteristics influence weight loss maintenance (Paixão et al., 2020).

To improve the use of successful weight management behaviors and promote habit formation, some studies showed that (1) adherence to a meal plan, (2) a good social support system, (3) limiting certain types of food or/and having healthy foods at home, (4) problem-solving abilities could be relevant, (5) exercise Motivation and self-efficacy, (6) reduced perceived barriers, and (7) weight management tools, such as calorie counting apps (Teixeira et al., 2009; Andrés et al., 2011; Kapoor et al., 2017; Cheng et al., 2018; Paixão et al., 2020). However, the available instruments, which evaluate weight management, focus only on a small set of possible approaches and spotlight strategies centered on behavior change interventions (Keller and Siegrist, 2015; Hartmann-Boyce et al., 2016). A recent systematic review reported that different groups of people (e.g., men vs. women, younger vs. older) employed different sets or combinations of cognitive and behavioral strategies to lose weight and this needs to be considered (Paixão et al., 2020).

The Oxford Food and Activity Behaviors (OxFAB) taxonomy and subsequent questionnaire were recently developed through qualitative analysis, based on existing weight management resources, behavior change theories (e.g., health action process approach), and previous taxonomies (e.g., behavior change technique taxonomy), systematizing the cognitive-behavioral strategies adopted by individuals who are attempting to manage their weight. The taxonomy consists of 117 questions grouped into 23 domains (Hartmann-Boyce et al., 2016). The questionnaire has already been applied in a cohort study, with 117 items organized into 21 domains: (1) Energy Compensation, (2) Goal Setting, (3) Imitation: Modeling, (4) Impulse Management: Acceptance, (5) Impulse Management: Awareness of Motives, (6) Impulse Management: Distraction, (7) Information Seeking, (8) Motivation, (9) Planning Content, (10) Regulation: Allowances, (11) Regulation: Restrictions, (12) Regulation: Rule Setting, (13) Restraint, (14) Reward, (15) Scheduling of Diet and Activity, (16) Self-Monitoring, (17) Stimulus Control, (18) Support: Buddying, (19) Support: Motivational, (20) Support: Professional Help, and (21) Weight Management Aids (Hartmann-Boyce et al., 2018).

Instead of using the previously developed questionnaire, the present study applied a new set of items (driven by the OxFAB taxonomy), which were developed by a team of five experts (including the author of the OxFAB Taxonomy and original questionnaire) and considering all the OxFAB domains to explore those that best represent the strategies employed, specifically by middle-aged women because:

(1) The cohort study by Hartmann-Boyce et al. (2018) focused on a sample with different characteristics from the present study, such as (a) younger and older population (age > 18 years), (b) both sexes (women and men), (c) only adults with overweight or obesity (BMI ≥ 25 kg/m^2^), and (d) participants purposely trying to lose weight. The present study entailed (a) middle-aged women (45–65 years), (b) unconstrained BMI, and (c) participants attempting and not attempting to lose weight.
(2) The sample of the previous cohort study was not representative, which limits the applicability of the findings across a range of population groups (Hartmann-Boyce et al., 2018). It is important to have particular attention to culture (e.g., the influence of cultural norms on food consumption) and sex specificity (Andrés et al., 2011; Paixão et al., 2020).
(3) Also, the original study (Hartmann-Boyce et al., 2016) only tested the validity and reliability of the measure. The present study tested the construct validity (factorial, convergent, and discriminant), criterion validity (obesity vs. normal weight), reliability, and external validity to assert the robustness of the instrument.
(4) In addition, the authors of the previous cohort study emphasized that the amount of missing data (60% of the participants were excluded) limited the generalizability of the conclusion (Hartmann-Boyce et al., 2018).
(5) Finally, generalizable weight management taxonomies are relevant for heterogeneous interventions. However, middle-aged women may require tailored interventions to prevent weight gain in this stage of reproductive aging. Consequently, it is essential to test and validate new measures based on what middle-aged women define as being meaningful and successful in weight management (Williams et al., 2007; Stacey et al., 2015; Melendez-Torres et al., 2018).

Given these arguments and the lack of knowledge about the preferred/most frequently used weight management strategies in middle-aged women (Molarius et al., 2020), this study sought to (1) develop an OxFAB questionnaire for middle-aged women (OxFAB-MAW) encompassing strategies from all original domains, (2) assess its psychometric properties (including reliability, factorial, convergent, discriminant, external, and criterion validities) in a specific health-related risk group (middle-aged women), and (3) evaluate the discriminative power of the tool among women with a healthy weight (18.5 kg/m^2^ ≥ BMI ≤ 25 kg/m^2^) and with clinical weight, that is, obesity (BMI ≥ 30 kg/m^2^).

## 2. Materials and methods

### 2.1. Participants

A minimum of 500 participants’ sample (10 participants per item) was desirable for this validation, given that an initial pool of 50 items was expected. A total of 1,993 middle-aged Portuguese women completed an online survey. The inclusion criteria were: (1) sex (only women), (2) nationality (Portuguese), (3) age (between 45 and 65 years), (4) literacy skills, and (5) internet access. The only exclusion criterion was: (1) non-Portuguese. Participants who reported a different nationality (e.g., other than Portuguese) were considered ineligible and excluded from the study (*n* = 72).

### 2.2. Ethical considerations

This study is part of the research project (ME-WEL: menopause and weight loss), which aims at contributing to the promotion of a healthier life and wellbeing for middle-aged women (specifically menopausal women), meeting the United Nations 2030 Agenda. The study was approved by the Ethics Committee of Ispa–Instituto Universitário (ref. D/024/01/2020) and followed the ethical guidelines and the standards of the American Psychological Association [APA] (2003) and the Portuguese Psychologist Association [PPA] (2011). Informed consent (including the main goal, procedures of the study, assuring confidentiality of the given information, and voluntary participation) was electronically provided to all participants, as well as the contact of the researcher, to answer any questions arising from the study and its legitimacy.

### 2.3. Procedures

First, the item generation process was conducted to determine the appropriate questions for each domain (Boateng et al., 2018). On the original OxFAB taxonomy, all domains were explained, and examples were added to aid comprehension (Hartmann-Boyce et al., 2016). The domains included different weight management behaviors, and these behaviors expressed (1) physical activity items, (2) food consumption items, and sometimes (3) other behaviors not necessarily related to food ingestion/physical activity, such as item 5 [“*How often d*o *you establish goals regarding weight loss? (e.g., aiming to wear a certain trouser size)*;” Goal Setting domain], item 8 [“*How often do you imitate other people’s weight management behavior? (e.g., did you weigh yourself weekly as you saw a family member, or a friend do?)*;” Imitation: Modeling domain], item 20 (“*How often do you seek information on how to manage your weight?*;” Information Seeking domain), item 21 [“*How often do you use strategies to motivate yourself toward weight loss? (e.g., monitoring your progress through charts/apps, looking at pictures of yourself with more/less weight to motivate yourself)*;” Motivation domain], item 33 [“W*hen you reach the goals you set for yourself, do you Reward yourself? (e.g., set aside some money to buy yourself a reward in case you achieve your goals)*;” Reward domain], item 36 (“*How often do you go to bed every night at the same hour?*;” Scheduling of Diet and Activity domain), item 39 [“*How often do you monitor your weight or measure your body shape? (e.g., assess how tight/large your clothes are, measure your waist with tape measure)*;” Self-Monitoring domain], item 46 [“*How often do you seek support or shared with others (e.g., family members and colleagues) your weight management plans or goals?*;” Support: Motivational domain], and item 47 [“*How often do you seek professional help to lose weight? (e.g., seek help to deal with feelings of sadness, anxiety, or stress; seek the help of a health professional to lose weight)*;” Support: Professional Help domain]. Six domains contained all three types of behaviors (food consumption, physical activity, and other behaviors with the impact on weight management), 11 domains included only two types of behaviors (physical activity and food ingestion), and six domains included only one type of behavior.

Since there were 117 strategies in the original instrument (Hartmann-Boyce et al., 2016), one of the goals of the present study was to reduce the number of items to facilitate its use in research and clinical settings. There was a special focus on the form and the wording of the items, especially on being simple to understand and unambiguous (Boateng et al., 2018). Although the cohort study included only 21 domains, the original taxonomy contemplated 23 domains, and thus the present study included items representing all these 23 domains (Hartmann-Boyce et al., 2016, 2018). In the end, item creation and response scale were reviewed by a panel of five experts (team members).

The importance of the response scale was also considered (Boateng et al., 2018): a 5-point Likert scale was chosen, ranging from 0 (*Never*) to 4 (*Always*), including the option *Not applicable to me*, as it was recommended in the OxFAB taxonomy to explore strategies that were not relevant to the participants. However, in the present study, all answers *Not applicable to me* were rated as missing data, as performed in other studies (Merz et al., 2018).

A non-probabilistic online sample was recruited between May and December 2020. This study was disseminated on social media (e.g., Facebook and Instagram), targeting platforms focused on women’s issues, menopause, weight, health, and groups organized by health professionals (e.g., nutritionists). The participants were also recruited by electronic email solicitation, addressed to several health-related institutions (e.g., medical centers).

### 2.4. Measures

All participants provided information (self-reported) about demographic factors (e.g., age and the highest level of education), health-related information (e.g., recent psychological problem), lifestyle factors (e.g., physical activity practice), and BMI [weight (kg)/height^2^]. Weight management strategies were assessed by the OxFAB-MAW, composed of 49 items, representing the 23 domains of the OxFAB taxonomy (Hartmann-Boyce et al., 2016). A higher score indicated more frequent use of the strategy, and consequently, of the domain.

The OxFAB taxonomy items had already been tested regarding their face validity, as well as reliability, through a test–retest (a mean PABAK score of 0.61; *SD* = 0.15) (Hartmann-Boyce et al., 2016).

### 2.5. Statistical analyses

Descriptive statistics [means (M), standard deviations (SD), medians (Me), skewness (Sk), and kurtosis (Ku)] were performed. The item’s sensibility, severe univariate normality, and homogeneity violations of normality were assessed using absolute values of sk > 3 and ku > 7 (Marôco, 2021). A confirmatory factor analysis (CFA), using the diagonally weighted least squares (WLSMV) estimation method, specifically designed for ordinal data, in the Lavaan package, was performed (Rosseel, 2012; Marôco, 2021). To determine model fit, comparative fit index (CFI), Tucker–Lewis index (TLI), root mean square error of approximation (RMSEA), and standardized root mean square residual (SRMR) were assessed. Values of CFI and TLI > 0.9, an RMSEA of <0.08 or 0.05 (confidence interval higher limit 90%, lower than 0.10), and an SRMR of <0.5 suggest an acceptable good model fit (Schermelleh-Engel et al., 2003; Marôco, 2021). Based on these model fit indices and other parameters and considerations (e.g., factorial loadings and theoretical research), the model was refined step by step according to modification indices (MIs) calculated with the Lagrange multipliers. Changes were made based on LM > 11 (*p* < 0.001) and theoretical considerations (Marôco, 2021).

Construct reliability was assessed by McDonalds’ Omega and the internal consistency (Cronbach’s alpha) using the semTools package (Jorgensen et al., 2019). Values of >0.6–0.7 were considered acceptable because it is a large sample study (Fornell and Larcker, 1981). However, higher results determined better internal consistency/reliability (Marôco, 2021). Construct validity was evaluated with convergent validity, that is, average variance extracted (AVE), which must be ≥ 0.5 for evidence of convergent validity (Fornell and Larcker, 1981). Discriminant validity was also estimated and assessed by comparing the AVE for each factor with the square of Pearson’s correlation between the factors. AVE greater than the square of the correlation between factors was considered evidence of discriminant validity (Fornell and Larcker, 1981).

Through a multigroup confirmatory factor analysis, external validity was tested, analyzing the measurement invariance of the final model in 50% of the sample (the sample was randomly selected—test group vs. validation sample). Configural, metric, scalar, and restrict invariances were tested (Marôco, 2021). Values, such as ΔCFI ≤ 0.01, ΔTLI ≤ 0.01, and ΔRMSEA ≤ 0.015, indicated that the model was invariant (Chen, 2007). In addition, to assess the differences between the non-clinical (normal weight group) and clinical group (obesity group), an independent *t*-test was performed to assert evidence of discriminant criterion validity.

All statistical analyses were performed using IBM SPSS Statistics (v. 27) and R-Lavaan (Rosseel, 2012), through RStudio (R Core Team, 2020; RStudio Team, 2020).

## 3. Results

### 3.1. Participants

The final participants included 1,921 women; however, participants who selected the option *Not applicable to me* (rated as missing data) were not considered and were excluded from the analyses. Thus, eligible participants’ (*n* = 1,367) characteristics are shown in Table 1. On average, participants were 52.3 years old (*SD* = 5.15). The average weight of the participants was 69.48 kg (*SD* = 13.21) and their BMI was 26.42 (*SD* = 4.77). Approximately 48% (*n* = 662) reported doing something to lose weight, such as physical activity or consuming healthy food.

**TABLE 1:** Characterization of participants.

	*n*	*%*
Age *(M, SD)*	*(52.3, 5.15)*	
**Relationship status**		
In a relationship, and living with the partner	959	70.2
In a relationship, but not living with the partner	143	10.5
Single	265	19.4
**Parity**		
No children	160	11.7
1 or 2 children	1042	76.3
3 or more children	165	12.1
**Highest education level obtained**		
Primary school	7	0.5
Middle school	25	1.8
High school	557	40.8
College degree	778	56.9
**Professional Status**		
Active	1133	82.9
Inactive (e.g., retired)	234	17.1
**Annual Household Income**		
Until 10.000€	243	17.8
Between 10.001 and 20.000€	395	28.9
Between 20.001 and 37.500€	440	32.2
Between 37.501 and 70.000€	217	15.9
Higher than 70.001€	72	5.3
**Recent disease**		
Yes	259	18.9
No	1108	81.1
**Recent psychological problem**		
Yes	226	16.5
No	1141	83.5
**Physical activity/exercise**		
Yes	907	66.3
No	460	33.7
**BMI status**		
Underweight (BMI < 18.5 kg/m^2^)	10	0.7
Normal weight (18.5 kg/m^2^ ≥ BMI < 25 kg/m^2^)	597	43.7
Overweight (25 kg/m^2^ ≥ BMI < 30 kg/m^2^)	480	35.1
Obesity (BMI ≥ 30 kg/m^2^)	280	20.5

### 3.2. Items’ distributional properties

Around 29% of the participants were excluded (*n* = 554), that is, participants who chose *Not applicable to me*. The average scores (min.: 0 – *Never*; max.: 4 – *Always*) for OxFAB-MAW items ranged from 0.58 (*SD* = 0.941) for item 37 (“*How often do you go to bed every night at the same hour?”*) to 1.90 (*SD* = 1.178) for item 26 (“*How often do you avoid buying or eating certain foods?* – e.g., *avoid eating foods you particularly like*”), and 0.00 < *Me* < 2.00. The OxFAB-MAW’s items did not show any sensitivity or severe non-normality problems (0.06 < *Sk* < 1.25; –1.02 < *Ku* < 2.41).

### 3.3. Construct validity: CFA, convergent, and discriminant validities

Since there were five domains with only one item – (1) Impulse Management: Delay (“*How often do you postpone/interrupt eating, when you have craving?*”), (2) Impulse Management: Substitution (“*How often do you replace eating with other activities to reduce the desire to eat? –* e.g., *go to sleep or brush your teeth*”), (3) Motivation (“*How often do you use strategies to motivate yourself toward weight loss? –* e.g., *monitoring your progress through charts/apps, looking at pictures of yourself with more/less weight to motivate yourself*”), (4) Reward (“*How often do you reward yourself, when you reach the goals you set for yourself? –* e.g., *set aside some money to buy yourself a reward in case you achieve your goals*”), and (5) Support: Professional Help (“*How often do you seek professional help to lose weight? –* e.g., *seek help to deal with feelings of sadness, anxiety, or stress; seek the help of a health professional to lose weight*”), these were grouped into other domains, using a theory-driven decision. Hence, the previous items were attributed to the following dimensions: (1) Impulse Management: Acceptance, (2) Impulse Management: Distraction, (3) Support: Motivational, (4) Goal Setting, and (5) Support: Help from Others, respectively (Table 2). Thus, instead of having a structure with 23 domains, the model was tested with 18 theory-driven domains.

**TABLE 2:** Initial and final tool of Oxford Food and Activity Behaviors taxonomy (OxFAB-MAW) domains and items.

Initial OxFAB-MAW domains and items	Final OxFAB-MAW domains and items
Energy Compensation – Items 1, 2	Energy Compensation – Items 1, 2
Goal Setting – Items 3, 4, 5	Goal Setting – Items 3, 4, 5, 33
Imitation: Modeling – Items 6, 7, 8	Imitation: Modeling – Items 6, 7, 8
Impulse Management: Acceptance – Items 9, 10, 11	Impulse Management: Acceptance – Items 9, 10, 11, 16
Impulse Management: Awareness of Motives – Items 12, 13	Impulse Management: Awareness of Motives – Items 12, 13
Impulse Management: Distraction – Items 14, 15	Impulse Management: Distraction – Items 14, 15, 17
Impulse Management: Delay – Item 16	Information Seeking – Items 18, 19, 20, 21
Impulse Management: Substitution – Item 17	Planning Content – Items 22, 23
Information Seeking – Items 18, 19, 20	Regulation: Allowances – Items 24, 25
Motivation – Item 21	Regulation: Restrictions – Items 26, 27, 28
Planning Content – Items 22, 23	Regulation: Rule Setting – Items 29, 30
Regulation: Allowances – Items 24, 25	Restraint – Items 31, 32
Regulation: Restrictions – Items 26, 27, 28	Scheduling of Diet and Activity – Items 34, 35, 36
Regulation: Rule Setting – Items 29, 30	Self-Monitoring – Items 37, 38, 39
Restraint – Items 31, 32	Stimulus Control – Items 40, 41
Reward – Item 33	Support: Help from Others – Items 42, 43, 47
Scheduling of Diet and Activity – Items 34, 35, 36	Support: Motivational – Items 44, 45, 46
Self-Monitoring – Items 37, 38, 39	
Stimulus Control – Items 40, 41	
Support: Buddying – Items 42, 43	
Support: Motivational – Items 44, 45, 46	
Support: Professional Help – Item 47	
Weight Management Aids – Items 48, 49	

The CFA was performed for the 18 first-order factors of the OxFAB-MAW in half of the sample, the group test (*n* = 960). Initially, the goodness-of-fit indices did not suggest an acceptable model fit [CFI = 0.916; TLI = 0.898; RMSEA = 0.076, P (RMSEA ≤ 0.05) < 0.001, 90% CI: 0.074; 0.078; SRMR = 0.059]; therefore, changes in the model were made as described next. The domain Weight Management Aids was removed because the values of AVE (0.35), ω (0.44), and Cronbach’s alpha (0.52) were not acceptable; as such, the model was restructured in 17 domains (excluding the Weight Management Aids domain, which entailed two items).

Regarding the other items, all standardized factor loadings were higher than 0.5, except for item 36 (Scheduling of Diet and Activity domain: “*How often do you go to bed every night at the same hour?*”), which showed a low factorial weight (λ = 0.48: Table 3). However, it was not removed because this is the only item that evaluates sleep quality, which is an important element in weight management (Kapoor et al., 2017). Based on modification indices analysis, item 21 (Support: Motivational domain: “*How often do you use strategies to motivate yourself toward weight loss? –* e.g., *monitoring your progress through charts/apps, looking at pictures of yourself with more/less weight to motivate yourself*”) was moved to the Information Seeking domain. The final model, with 17 domains and 47 items, demonstrated an acceptable fit [CFI = 0.928; TLI = 0.913; RMSEA = 0.072, P (RMSEA ≤ 0.05) < 0.001, 90% CI: 0.070; 0.075; SRMR = 0.054].

**TABLE 3:** OxFAB-MAW domains, items, and factor loadings.

Domain	Items	Factor loading
Energy Compensation	Item 1: How often throughout the day, do you adjust what you eat according to how much you will eat/have eaten or will exercise/have exercised (e.g., if I have already eaten too much today, I will eat less the rest of the day).	0.83
Energy Compensation	Item 2: How often throughout the day, do you adjust your physical activity according to how much you will eat/have eaten or will exercise/have exercised (e.g., if I have already eaten too much today, I will work out more).	0.88
Goal Setting	Item 3: How often do you establish goals regarding food ingestion? (e.g., quantity, type of food, etc.).	0.82
Goal Setting	Item 4: How often do you establish goals regarding physical activity? (e.g., days for practicing physical activity, duration).	0.76
Goal Setting	Item 5: How often do you establish goals regarding weight loss? (e.g., aiming to wear a certain trouser size).	0.78
Imitation: Modeling	Item 6: How often do you imitate other people’s dietary behavior? (e.g., copying the diet behavior of family members or friends).	0.84
Imitation: Modeling	Item 7: How often do you imitate other people’s physical activity behavior? (e.g., copying the physical activity behavior of family members or friends).	0.94
Imitation: Modeling	Item 8: How often do you imitate other people’s weight management behavior? (e.g., did you weigh yourself weekly as you saw a family member, or a friend do)?	0.86
Impulse Management: Acceptance	Item 9: How often do you accept feelings of hunger when they come? (e.g., accepted that feeling and acted on it).	0.70
Impulse Management: Acceptance	Item 10: How often do you accept cravings when they come? (e.g., accepted these cravings and did not eat).	0.79
Impulse Management: Acceptance	Item 11: How often do you accept uncomfortable aspects of physical activity? (e.g., tolerated the pain or sweating).	0.78
Impulse Management: Awareness of Motives	Item 12: How often do you ask yourself if you’re hungry when you feel like eating or when you’re already having a meal?	0.84
Impulse Management: Awareness of Motives	Item 13: How often do you ask yourself why you don’t feel like practicing physical activity?	0.82
Impulse Management: Distraction	Item 14: How often do you find another activity to do when you feel like eating?	0.82
Impulse Management: Distraction	Item 15: When you feel uncomfortable practicing physical activity, do you distract yourself with something?	0.78
Impulse Management: Acceptance	Item 16: When you have a craving, do you postpone/interrupt food ingestion?	0.78
Impulse Management: Distraction	Item 17: How often do you replace further food ingestion with other activities to reduce the desire to eat? (e.g., go to sleep or brush your teeth).	0.82
Information Seeking	Item 18: How often do you seek information on food components/calories?	0.82
Information Seeking	Item 19: How often do you seek information on the calories you burn during physical activity?	0.86
Information Seeking	Item 20: How often do you seek information on how to manage your weight?	0.90
Information Seeking	Item 21: How often do you use strategies to motivate yourself toward weight loss? (e.g., monitoring your progress through charts/apps, looking at pictures of yourself with more/less weight to motivate yourself).	0.90
Planning Content	Item 22: How often do you plan what you’re going to eat throughout your day? (e.g., bringing food from home; preparing a shopping list; carrying healthy snacks).	0.80
Planning Content	Item 23: How often do you plan the physical activity you’re going to do throughout your day? (e.g., incorporate moments for physical activity on your way to work; explore types of physical activity you might like; create a workout plan).	0.83
Regulation: Allowances	Item 24: How often do you allow yourself to eat unlimited amounts of certain foods/drinks? (e.g., allow yourself to eat/drink whatever you want after a period of restriction; allow yourself a “cheat” day/meal or a day/meal out of the usual dietary plan).	0.76
Regulation: Allowances	Item 25: How often do you allow yourself to practice less intense physical activity (or not to practice any kind of physical activity on a given day), after a few days of regular physical activity?	0.91
Regulation: Restrictions	Item 26: How often do you avoid buying or eating certain foods? (e.g., avoid eating foods you particularly like).	0.87
Regulation: Restrictions	Item 27: How often do you avoid eating at certain hours of the day? (e.g., skip the last meal of the day or eat a smaller quantity).	0.83
Regulation: Restrictions	Item 28: How often do you avoid going out with friends/colleagues or certain places (e.g., restaurants) to avoid eating certain foods?	0.76
Regulation: Rule Setting	Item 29: How often do you establish rules to regulate what you eat? (e.g., eat slowly; leave the table once you’ve finished eating; not eating everything on your plate).	0.80
Regulation: Rule Setting	Item 30: How often do you maximize the potential for physical activity whilst active? (e.g., walk the longest route whenever you walk to work; walk at a faster pace instead of walking slowly).	0.78
Restraint	Item 31: How often do you limit your diet, in a rigid way? (e.g., not ingesting several foods with the aim of controlling or losing weight).	0.86
Restraint	Item 32: How often do you limit the physical activity you do, in a rigid way? (e.g., constantly practicing only one type of physical activity because you believe it’s the best one to manage your weight).	0.80
Goal Setting	Item 33: When you reach the goals you set for yourself, do you reward yourself? (e.g., set aside some money to buy yourself a reward in case you achieve your goals).	0.75
Scheduling of Diet and Activity	Item 34: How often do you establish schedules for having meals or shopping for food? (e.g., eat at certain times, even if you’re not hungry; eating before going shopping to not shop hungry).	0.81
Scheduling of Diet and Activity	Item 35: How often do you establish schedules for physical activity (e.g., give up something else to have time for physical activity; practice physical activity at a time when there are less people).	0.82
Scheduling of Diet and Activity	Item 36: How often do you go to bed every night at the same hour?	0.48
Self-Monitoring	Item 37: How often do you measure or weigh what you eat? (e.g., write down calories, weigh foods).	0.76
Self-Monitoring	Item 38: How often do you measure the amount of physical activity you practice? (e.g., write down the amount of time dedicated).	0.79
Self-Monitoring	Item 39: How often do you monitor your weight or measure your body shape? (e.g., assess how tight/large your clothes are, measure your waist with tape measure).	0.78
Stimulus Control	Item 40: How often do you limit the available food quantities to better control what you eat? (e.g., eat from a smaller plate/bowl; buy less quantity of food; not having unhealthy foods/beverages at home).	0.78
Stimulus Control	Item 41: How often do you give yourself cues for being physically active or to increase the amount of physical activity? (e.g., set an alarm to get up and walk a little every 2 h).	0.77
Support: Help from others	Item 42: How often do you diet with someone who is also dieting? (e.g., friend, family member).	0.76
Support: Help from others	Item 43: How often do you exercise with someone who is also exercising? (e.g., friend, family member).	0.65
Support: Motivational	Item 44: How often do you ask people around you not to offer you foods you are trying to avoid?	0.80
Support: Motivational	Item 45: How often do you participate in physical activity groups?	0.66
Support: Motivational	Item 46: How often do you seek support or shared with others (e.g., family members, colleagues) your weight management plans or goals?	0.83
Support: Help from others	Item 47: How often do you seek professional help to lose weight? (e.g., seek help to deal with feelings of sadness, anxiety, or stress; seek the help of a health professional to lose weight).	0.69

Almost all domains showed good convergent validity (i.e., AVE ≥ 0.50), ranging from 0.52 (Scheduling of Diet and Activity) to 0.79 (Imitation: Modeling; see Table 4); the exception was the Support: Help from Others domain, which showed borderline convergent validity (AVE = 0.49). Discriminant validity was tested, comparing the AVE of each factor with the inter-factors’ squared correlation. Of the 136 paired factors comparisons, 93 presented discriminant validity.

**TABLE 4:** Average variance extracted and reliability analysis of OxFAB-MAW.

	EC	GS	IM	IM(A)	IM(AM)	IM(D)	IS	PC	R(A)	R(R)	R(RS)	R	SDA	SM	SC	S(HO)	S(M)
α	0.84	0.83	0.92	0.83	0.82	0.85	0.92	0.80	0.82	0.83	0.77	0.82	0.74	0.81	0.75	0.73	0.80
ω	0.83	0.78	0.88	0.82	0.78	0.81	0.90	0.76	0.79	0.82	0.73	0.78	0.71	0.76	0.69	0.67	0.75
AVE	0.73	0.61	0.79	0.58	0.69	0.65	0.75	0.67	0.70	0.67	0.63	0.70	0.52	0.60	0.59	0.49	0.58

α, Cronbach’s alphas; ω, McDonalds’ omega; AVE, average variance extracted; EC, Energy Compensation; GS, Goal Setting; I(M), Imitation: Modeling; IM(A), Impulse Management: Acceptance; IM(AM), Impulse Management: Awareness of Motives; IM(D), Impulse Management: Distraction; IS, Information Seeking; PC, Planning Content; R(A), Regulation: Allowances; R(R), Regulation: Restrictions; R(RS), Regulation: Rule Setting; R, Restraint; SDA, Scheduling of Diet and Activity; SM, Self-Monitoring; SC, Stimulus Control; S(HO), support: help from others; S(M), Support: Motivational.

### 3.4. Reliability: Internal consistency and composite reliability

All dimensions revealed an acceptable to good reliability regarding internal consistency (Cronbach’s alpha) and composite reliability (McDonalds’ Omega) (0.73 < α < 0.92 and 0.67 < ω < 0.90), as shown in Table 4.

### 3.5. External validity and discriminant criterion validity

The model tested in the second group (validation group; *n* = 982) presents an acceptable fit [CFI = 0.928; TLI = 0.913; RMSEA = 0.076; P (RMSEA ≤ 0.05) < 0.001, 90% CI: 0.074; 0.078; SRMR = 0.054]. The model showed external validity through a multigroup confirmatory analysis (ΔCFI < 0.009; ΔRMSEA < 0.000; Table 5).

**TABLE 5:** External validity, through multigroup confirmatory analysis.

Model	*X* ^2^	*Df*	*X^2^/df*	*p*	CFI robust	RMSEA robust	ΔCFI	ΔRMSEA
Configural	7567.6	1796	4.21		0.928	0.074		
Thresholds	7629.8	1920	3.97	0.602	0.929	0.071	0.001	−0.003
Metric	7661.7	1950	3.93	0.777	0.931	0.069	0.003	−0.002
Scalar	7661.7	1950	3.93		0.931	0.069	0.000	0.000
Means	7705.4	1967	3.92	0.568	0.940	0.064	0.009	−0.005

χ^2^, chi-square; *Df*, degrees of freedom; *p*, statistical significance.

In addition, to explore if the OxFAB-MAW instrument discriminated differences between the non-clinical (normal weight group, *n* = 597) and the clinical groups (obesity group, *n* = 280), discriminant criterion validity was assessed. These two groups were compared through an independent sample *t*-test to explore the OxFAB-MAW domains’ capacity to discriminate weight management behaviors in middle-aged women with two classes of weight (healthy weight and obesity). In addition, both validity (0.43 < AVE > 0.81) and reliability (0.66 < α < 0.93, and 0.62 < ω < 0.89) were asserted in these two specific groups. The model presents a good fit (CFI = 0.932; TLI = 0.918; RMSEA = 0.032; SRMR = 0.052) in both groups (non-clinical and clinical groups). Finally, the measure showed discriminant criterion validity in 11 of 17 domains (*p* < 0.05); moreover, in the domains with criterion validity, all means were higher in the clinical group, as shown in Table 6.

**TABLE 6:** Criterion validity (normal group vs. clinical group) of OxFAB-MAW, through an independent *T*-test.

Domain	*df*	*t*	*P*	*I.C.*	Cohen’s *d*	Normal weight group (*n* = 597)		Obesity group (*n* = 280)	
						*M*	*SD*	*M*	*SD*
EC	622.54	0.168	0.866	[−0.13; 0.15]	0.012	1.44	1.087	1.43	0.942
GS	598.29	−1.179	0.239	[−0.21; 0.05]	−0.082	1.34	0.964	1.41	0.873
I(M)	499.11	−5.985	<0.001	[−0.50; −0.25]	−0.450	0.60	0.810	0.98	0.897
IM(A)	591.00	−2.126	0.034	[−0.26; −0.01]	−0.149	1.49	0.934	1.62	0.856
IM(AM)	875	−2.623	0.009	[−0.34; −0.05]	−0.190	1.40	1.030	1.59	0.979
IM(D)	875	−4.411	<0.001	[−0.42; −0.16]	−0.320	1.13	0.929	1.42	0.903
IS	875	−3.968	<0.001	[−0.47; −0.16]	−0.287	1.38	1.095	1.69	1.085
PC	875	−1.496	0.135	[−0.28; 0.04]	−0.108	1.47	1.122	1.59	1.088
R(A)	610.78	−0.912	0.362	[−0.20; 0.07]	−0.063	1.42	1.052	1.48	0.931
R(R)	875	−3.949	<0.001	[−0.40; −0.14]	−0.286	1.36	0.954	1.63	0.927
R(RS)	875	−0.986	0.325	[−0.21; 0.07]	−0.071	1.28	1.006	1.35	0.985
R	875	−3.337	0.001	[−0.38; −0.10]	−0.242	1.07	1.002	1.31	0.945
SDA	875	−1.199	0.231	[−0.21; 0.05]	−0.087	1.34	0.922	1.42	0.934
SM	875	−2.489	0.013	[−0.29; −0.03]	−0.180	0.87	0.906	1.04	0.884
SC	875	−3.176	0.002	[−0.37; −0.09]	−0.230	0.96	0.988	1.19	1.018
S(HO)	875	−5.313	<0.001	[−0.44; −0.20]	−0.385	0.83	0.811	1.15	0.902
S(M)	875	−3.168	0.002	[−0.33; −0.08]	−0.229	0.79	0.865	0.99	0.907

*df*, degrees of freedom; *t*, t-test; *p*, statistical significance; *I.C.*, 95% confidence interval of the difference; *M*, mean; *SD*, standard deviations; EC, Energy Compensation; GS, Goal Setting; I(M), Imitation: Modeling; IM(A), Impulse Management: Acceptance; IM(AM), Impulse Management: Awareness of Motives; IM(D), Impulse Management: Distraction; IS, Information Seeking; PC, Planning Content; R(A), Regulation: Allowances; R(R), Regulation: Restrictions; R(RS), Regulation: Rule Setting; R, Restraint; SDA, Scheduling of Diet and Activity; SM, Self-Monitoring; SC, Stimulus Control; S(HO), support: help from others; S(M), Support: Motivational.

## 4. Discussion

Effective weight management strategies are an emergent variable in the literature, and theory-driven valid and reliable instruments are necessary to assess them (Kapoor et al., 2017; Proietto, 2017; Melendez-Torres et al., 2018). Also, given the high rate of obesity among middle-aged women, it is crucial to explore this specific risk group to provide enhanced scientific insight (Kapoor et al., 2017; Cheng et al., 2018; Schreiber and Dautovich, 2018). In this regard, the present study describes the development of an OxFAB-based assessment tool in Portuguese, adapted to middle-aged women (OxFAB-MAW), and an evaluation of the psychometric properties and the discriminative power of the tool among women with a healthy weight (18.5 kg/m^2^ ≥ BMI ≤ 25 kg/m^2^) and clinical weight (obesity; BMI ≥ 30 kg/m^2^).

The results suggest that the sensitivity, construct validity, and reliability of OxFAB-MAW were acceptable. The structural stability of OxFAB-MAW was confirmed, that is, the measurement model had external validity, showing configural, metric, scalar, and mean invariances, and also discriminant criterion validity (normal weight vs. obesity group) in 11 of 17 domains. The responses *Not applicable to me* were excluded since some studies showed that this option scoring as 0 yields statistical problems when models are judged by stringent criteria (Boateng et al., 2018; Merz et al., 2018). Nonetheless, the sample was large enough to support all planned analyses, and the robustness of the information, that is, considering only the frequency (never-always) of the participants’ strategies —revealed more clinically pertinent information (based on what middle-aged women actually do to manage their weight).

### 4.1. Confirmatory factor analysis and discriminant validity

Initially, five domains with only one item were grouped into other domains. First, Impulse Management: Delay was integrated into Impulse Management: Acceptance, since the delay subdomain refers to postponing a behavior (in this case, the ingestion of a specific food) when there is an urge to do so, and the acceptance subdomain defines a response (e.g., an acceptance) to manage the impulses (or urges) that arise (Hartmann-Boyce et al., 2017). In addition, the Impulse Management: Substitution domain, which is related to the use of alternative behaviors (e.g., brushing teeth) to avoid the ingestion of a particular food, was combined with the Impulse Management: Distraction domain, which refers to the moment when an urge to ingest a particular food arises. These two complemented each other since one of the responses to manage the impulse is a distraction behavior (e.g., go to sleep; Hartmann-Boyce et al., 2016, 2017). Support from others (health professionals and friends/family) can promote a higher Motivation for behavioral change (e.g., healthy eating and consequent weight loss) in women (especially with a higher BMI; Gettens et al., 2018). Other authors identified extrinsic Motivation as related to supportive relationships (Melendez-Torres et al., 2018). For that reason, the only Motivation item in the questionnaire was allocated to the Support: Motivational domain. Furthermore, this domain is defined, according to the original study, as a “*discussing, pledging, or revealing weight loss goals, plans, or achievements, or challenges to others to bolster Motivation*” (p. 318; Hartmann-Boyce et al., 2016). The only Reward item was placed in the Goal Setting domain because some studies demonstrated that they are strongly associated. For example, the person who wants to maintain/lose weight can be more focused to achieve his/her goals when they are associated with a reward, either short term or long term (Woolley and Fishbach, 2017). Finally, the Support: Professional Help item went to Support: Help from Others because both domains are correlated, and women who express the intention to lose weight frequently find both formal (e.g., professional help) and informal support important (e.g., buddying support; Molarius et al., 2020).

To improve the original model fit, two changes were made. The domain Weight Management Aids (item 48: “*How often do you use meal replacements to control your weight?*” and item 49: “*How often do you use exercise equipment? – e.g., at home or pay to go to a gym*”) was removed because some values were not acceptable. According to the OxFAB Taxonomy and cohort studies, this domain was one of the least used by people (such as in this study), and people who reported using this type of strategy were men and younger participants (Hartmann-Boyce et al., 2016, 2018). Second, item 21 (originally in the Motivation domain and moved to Support: Motivational domain: “*How often do you use strategies to motivate yourself toward weight loss? – e.g., monitoring your progress through charts/apps, looking at pictures of yourself with more/less weight to motivate yourself”*) was allocated to the Information Seeking domain, according to the modification indices. Item 36 (Scheduling of Diet and Activity domain: “*How often do you go to bed every night at the same hour?*”) showed a low factorial weight; however, it was not removed because it was the only item that evaluated sleep quality. According to some studies, in middle-aged women, sleep disturbance affects weight gain, and it is an important factor in weight management in this specific phase of life (Kapoor et al., 2017). Another study that evaluated middle-aged women’s needs when making body weight management decisions revealed that the importance of sleep should be considered (Stacey et al., 2015). After these changes, the measurement model presented an acceptable fit for this sample.

In the present study, some subscales did not show discriminant validity, but this was expected since all domains measure the same main construct, such as weight management strategies. Some behaviors were interdependent, so there may be a convergence of strategies’ implementation (when one occurs, so do some others), which might explain strong correlations among several domains (Paixão et al., 2020). Nonetheless, most domains presented discriminant validity, strengthening that the domains, although interdependent, reflect different behaviors (and distinct constructs as stated in the OxFAB Taxonomy), as mentioned earlier.

### 4.2. Criterion validity

In this study, criterion validity was performed. The OxFAB-MAW was shown to be sufficiently accurate to discriminate between a clinical group (with obesity) and a non-clinical group (normal weight), regarding several domains. Although some domains did not present criterion validity (Energy Compensation; Goal Setting; Planning Content; Regulation: Allowances; Regulation: Rule Setting; Scheduling of Diet and Activity); others, namely, (1) Imitation: Modeling, (2) Impulse Management: Acceptance, (3) Impulse Management: Awareness of Motives, (4) Impulse Management: Distraction, (5) Information Seeking, (6) Regulation: Restrictions, (7) Restraint, (8) Self-Monitoring, (9) Stimulus Control, (10) Support: Help from Others, and (11) Support: Motivational showed a discriminative power between the two different samples, and women with obesity performed all of them more frequently than counterparts with normal weight. This sample was recruited during the current COVID-19 pandemic, during both periods of national mandatory lockdown and not-in-lockdown. Further studies comparing the findings of the present study with those after the pandemic has ended might shed light on similarities and differences in weight management strategies between the two periods.

The domains, which did not discriminate between these two samples (women with obesity vs. normal weight), were mostly related to planning and goal-setting strategies, which seem to be associated with time perspective (future focus; Guthrie et al., 2014). The domain Regulation: Allowances also did not discriminate between samples. It may be that women in the normal weight range were using these weight management behaviors (though not frequently) to maintain or lose weight–this warrants further investigation.

The most used domains were explored in detail in the two distinct groups [normal weight (*n* = 597) vs. obesity (*n* = 280) groups]. It may be concluded that the most applied domains in the normal weight group were Impulse Management: Acceptance (*M* = 1.49; *SD* = 0.934) and Planning Content (*M* = 1.47; *SD* = 1.122), and in the obesity group, most applied domains were Information Seeking (*M* = 1.69; *SD* = 1.085) and Regulation: Restrictions (*M* = 1.63; *SD* = 0.927). Some studies showed that the Planning Content domain is associated with greater weight loss/maintenance, and in fact, in the present study, it was one of the most used domains in the normal weight sample (Hartmann-Boyce et al., 2017; Paixão et al., 2020). In contrast, the Impulse Management: Acceptance domain (one of the most applied domains in the normal weight group) is not the most used in the original (OxFAB Taxonomy) and cohort studies (Hartmann-Boyce et al., 2016, 2018).

In the present sample, the average weight of the participants was 69.5 kg (*SD* = 13.21), and BMI was 26.62 (*SD* = 5.71), much lower than the original study (the mean weight was 96.8 kg; *SD* = 21.0) and in the cohort study (the mean weight was 91.37 kg; *SD* = 18.38, whereby the BMI mean was 33.14; *SD* = 6.52; Hartmann-Boyce et al., 2016, 2018). Parallel to this evidence, the fact that Impulse Management: Acceptance is the most applied strategy might be related to the mainstream/social media dissemination of mindfulness-oriented interventions/behaviors (Michalak et al., 2020). Also, it might be postulated that an acceptance-based strategy facilitates flexibility development, and subsequently, more effective self-regulation of eating behavior. The acceptance is associated with lower impulsivity and may be related to more psychological flexibility (e.g., tolerating the physical sensations and impulses while not acting on them), being a stronger predictor of weight loss (Teixeira et al., 2009; Hartmann-Boyce et al., 2016).

It is important to emphasize that the average use of the strategies accounted for in the present instrument was low in both groups. This could be related to the fact that in the normal weight sample, 63.3% were not trying to lose weight (as expected). However, in the obesity sample, although 64.3% were trying to lose weight, the frequency of the strategies was very low.

Our results suggested a final questionnaire with 17 domains and 47 items, adapted to middle-aged women, available in Portuguese but which could be back-translated to English. The high number of items in the final instrument might be demanding for participants to answer in a short period of time. As such, a short version of OxFAB is currently in development.

The OxFAB-MAW proved to be a valid and reliable tool for evaluating weight management strategies. The fact that OxFAB-MAW is based on a widely known theory-driven taxonomy that focuses on the most frequent weight loss strategies makes this instrument useful for both clinical and research contexts addressing weight management with middle-aged women.

### 4.3. Strengths and limitations

Some limitations should be taken into account, namely, (1) due to the COVID-19 pandemic, the sample collection was online without a researcher’s presence (although if anyone had doubts, the only way to clear them up was by sending an email). (2) Since the sample was collected online, the women included were only those who had access to technology and had digital literacy, which may have biased some results. (3) We relied on self-reported weight, which, despite being the most frequently used measure, can lead to an underestimation of weight/height, and consequently, BMI. (4) The results may not be population representative since a non-probabilistic sample collection method was used. Nonetheless, (i) the present study entails a large sample of participants of a homogenous risk group (women between 45 and 65 years) and (ii) several analyses performed (including the external validity) support the conclusion that this instrument will have similar psychometric behavior in future samples with similar characteristics. Finally, (5) the study was performed during the exceptional condition of the new viral pandemic. Therefore, new studies are needed to confirm these results after the pandemic period.

Our research has some strengths that should be mentioned. The present instrument (OxFAB-MAW) assesses for the first time a wide variety of behaviors among middle-aged Portuguese women, which is a risk group for weight increase. Moreover, given the results of the present study, this age/sex group is not frequently involved with weight management strategies. This emphasizes the need to further explore these protective behaviors and develop interventions delivered in primary care settings to promote effective weight management adapted to mid-life women. Moreover, the psychometric qualities of this tool, and the fact it is theory-driven, make the OxFAB-MAW a good assessment in mapping the weight management behaviors in middle-aged women.

## 5. Conclusion

This study analyzed the psychometric properties of the OxFAB-MAW in a large middle-aged Portuguese sample, with two different groups (normal weight *vs.* clinical group – obesity). Despite the study’s limitations, the results provide strong support for the reliability and validity of OxFAB-MAW, which includes 17 domains and 47 items. Furthermore, this tool can discriminate between groups (normal weight vs. obesity group) in several OxFAB domains. This instrument can be helpful for evaluating more effective weight loss interventions, as it explores in detail several weight management strategies that, as the literature highlights, can be important in weight loss and subsequent success in maintaining that weight.

## Data availability statement

The raw data supporting the conclusions of this article will be made available by the authors, above reasonable request.

## Ethics statement

The studies involving human participants were reviewed and approved by the Ethics Committee of ISPA – Instituto Universitário (ref. D/024/01/2020). The patients/participants provided their written informed consent to participate in this study.

## Author contributions

ML was the principal investigator. ML and FP conducted the project development and the process of item generation. JH-B reviewed and approved the manuscript. MY performed the online survey, data collection, and data analysis. FP and JM reviewed the data analysis procedures. ML wrote the original draft, which was fully reviewed by FP, JM, JH-B, and FP-L. JH-B and FP-L did the English editing. All authors approved the submitted version.
