# Supplementary material for: Weight management strategies in Middle-Aged Women (MAW): Development and validation of a questionnaire based on the Oxford Food and Activity Behaviors Taxonomy (OxFAB-MAW) in a Portuguese sample
Source: Front Psychol. 2023 Jan 4;13:1069775. doi: 10.3389/fpsyg.2022.1069775 (PMC9846507; doi:10.3389/fpsyg.2022.1069775)
Supplement: Supplementary file 1 [file Table_1.DOCX]

**Appendices**

**OxFAB Instrument (In English)**

In the following section, you will find different questions about eating behaviour, physical activity, and weight management strategies. For each question, please place a cross (X) in the option that best suits your situation, in the last 30 days.

0 - Never

1 - Rarely

2 - Sometimes

3 - Often

4 - Always

**How often...**

1. Throughout the day, do you adjust what you eat according to how much you will eat/have eaten or will exercise/have exercised (e.g., if I have already eaten too much today, I will eat less the rest of the day).

2. Throughout the day, do you adjust your physical activity according to how much you will eat/have eaten or will exercise/have exercised (e.g., if I have already eaten too much today, I will work out more).

3. Do you establish goals regarding food ingestion? (e.g., quantity, type of food, etc.).

4. Do you establish goals regarding physical activity? (e.g., days for practicing physical activity, duration).

5. Do you establish goals regarding weight loss? (e.g., aiming to wear a certain trouser size).

6. Do you imitate other people’s dietary behaviour? (e.g., copying the diet behaviour of family members or friends).

7. Do you imitate other people’s physical activity behaviour? (e.g., copying the physical activity behaviour of family members or friends).

8. Do you imitate other people’s weight management behaviour? (e.g., did you weigh yourself weekly as you saw a family member, or a friend do)?

9. Do you accept feelings of hunger when they come? (e.g., accepted that feeling and acted on it).

10. Do you accept cravings when they come? (e.g., accepted these cravings and did not eat).

11. Do you accept uncomfortable aspects of physical activity? (e.g., tolerated the pain or sweating).

12. Do you ask yourself if you’re hungry when you feel like eating or when you’re already having a meal?

13. Do you ask yourself why you don’t feel like practicing physical activity?

14. Do you find another activity to do when you feel like eating?

15. When you feel uncomfortable practicing physical activity, do you distract yourself with something?

16. When you have a craving, do you postpone/interrupt food ingestion?

17. Do you replace further food ingestion with other activities to reduce the desire to eat? (e.g., go to sleep or brush your teeth).

18. Do you seek information on food components/calories?

19. Do you seek information on the calories you burn during physical activity?

20. Do you seek information on how to manage your weight?

21. Do you use strategies to motivate yourself towards weight loss? (e.g., monitoring your progress through charts/apps, looking at pictures of yourself with more/less weight to motivate yourself).

22. Do you plan what you’re going to eat throughout your day? (e.g., bringing food from home; preparing a shopping list; carrying healthy snacks).

23. Do you plan the physical activity you’re going to do throughout your day? (e.g., incorporate moments for physical activity on your way to work; explore types of physical activity you might like; create a workout plan).

24. Do you allow yourself to eat unlimited amounts of certain foods/drinks? (e.g., allow yourself to eat/drink whatever you want after a period of restriction; allow yourself a “cheat” day/meal or a day/meal out of the usual dietary plan).

25. Do you allow yourself to practice less intense physical activity (or not to practice any kind of physical activity on a given day), after a few days of regular physical activity?

26. Do you avoid buying or eating certain foods? (e.g., avoid eating foods you particularly like).

27. Do you avoid eating at certain hours of the day? (e.g., skip the last meal of the day or eat a smaller quantity).

28. Do you avoid going out with friends/colleagues or certain places (e.g., restaurants) to avoid eating certain foods?

29. Do you establish rules to regulate what you eat? (e.g., eat slowly; leave the table once you’ve finished eating; not eating everything on your plate).

30. Do you maximize the potential for physical activity whilst active? (e.g., walk the longest route whenever you walk to work; walk at a faster pace instead of walking slowly).

31. Do you limit your diet, in a rigid way? (e.g., not ingesting several foods with the aim of controlling or losing weight).

32. Do you limit the physical activity you do, in a rigid way? (e.g., constantly practicing only one type of physical activity because you believe it’s the best one to manage your weight).

33. When you reach the goals you set for yourself, do you reward yourself? (e.g., set aside some money to buy yourself a reward in case you achieve your goals).

34. Do you establish schedules for having meals or shopping for food? (e.g., eat at certain times, even if you’re not hungry; eating before going shopping to not shop hungry.

35. Do you establish schedules for physical activity (e.g., give up something else to have time for physical activity; practice physical activity at a time when there are less people).

36. Do you go to bed every night at the same hour?

37. Do you measure or weigh what you eat? (e.g., write down calories, weigh foods).

38. Do you measure the amount of physical activity you practice? (e.g., write down the amount of time dedicated).

39. Do you monitor your weight or measure your body shape? (e.g., assess how tight/large your clothes are, measure your waist with tape measure).

40. Do you limit the available food quantities to better control what you eat? (e.g., eat from a smaller plate/bowl; buy less quantity of food; not having unhealthy foods/beverages at home).

41. Do you give yourself cues for being physically active or to increase the amount of physical activity? (e.g., set an alarm to get up and walk a little every 2 hours).

42. Do you diet with someone who is also dieting? (e.g., friend, family member).

43. Do you exercise with someone who is also exercising? (e.g., friend, family member).

44. Do you ask people around you not to offer you foods you are trying to avoid?

45. Do you participate in physical activity groups?

46. Do you seek support or shared with others (e.g., family members, colleagues) your weight management plans or goals?

47. Do you seek professional help to lose weight? (e.g., seek help to deal with feelings of sadness, anxiety, or stress; seek the help of a health professional to lose weight).

**OxFAB Instrument (In Portuguese)**

De seguida, vai encontrar diferentes estratégias de gestão de peso. Avalie o quão frequentemente implementou cada uma destas estratégias nos últimos 30 dias. Há cinco hipóteses de resposta, escolha a alternativa que melhor se aplica a si.

0 - Nunca

1 - Raramente

2 – Às vezes

3 - Frequentemente

4 - Sempre

**Quão frequentemente...**

1. Ajustou, durante o dia, aquilo que comeu em função do que iria comer/comeu ou iria exercitar/exercitou (ex., se comeu muito num determinado momento do dia, comeu menos durante o resto do dia)?

2. Ajustou, durante o dia, a atividade física que fez em função do que iria comer/comeu ou iria exercitar/exercitou (ex., se comeu muito num determinado dia, fez mais atividade física)?

3. Estabeleceu objetivos em relação àquilo que comeu (ex., quantidades, tipo de comida, etc.)?

4. Estabeleceu objetivos de atividade física (ex., dias para fazer atividade física, duração)?

5. Estabeleceu objetivos relacionados com perda de peso (ex., definiu como meta vestir um determinado número de calças)?

6. Imitou o comportamento de dieta de outras pessoas (ex., dieta feita por familiares ou amigos)?

7. Imitou o comportamento de atividade física de outras pessoas (ex., atividade física feita por familiares ou amigos)?

8. Imitou o comportamento de controlo de peso de outras pessoas (ex., pesou-se semanalmente tal como viu fazer um familiar ou um amigo)?

9. Aceitou a sensação de fome, quando ela surgiu (ex., aceitou essa sensação e agiu de acordo com as suas necessidades)?

10. Aceitou um forte desejo *(cravings*) para comer, quando ele surgiu (ex., aceitou esse forte desejo e não comeu)?

11. Aceitou aspetos desconfortáveis da atividade física (ex., tolerou a dor ou a sudação associadas)?

12. Perguntou a si própria se estava com fome, quando lhe apeteceu comer ou quando já estava a fazer uma refeição?

13. Perguntou a si própria o porquê de não lhe apetecer fazer atividade física?

14. Encontrou outra atividade para fazer quando lhe apeteceu comer?

15. Distraiu-se com algo, quando se sentiu desconfortável enquanto fazia atividade física?

16. Interrompeu ou adiou comer algo, apesar de ter um forte desejo (*craving*) para o comer?

17. Fez outras atividades, em vez de comer mais, para reduzir o desejo de comer (ex., foi lavar os dentes ou foi dormir)?

18. Procurou informação acerca dos componentes ou calorias dos alimentos?

19. Procurou informação acerca das calorias que queimou durante a atividade física?

20. Procurou informação acerca de como gerir o seu peso?

21. Usou estratégias para se motivar a si própria para perder peso (ex., monitorizou progressos através de gráficos/apps, viu fotografias em que estava com mais ou menos peso para se motivar)?

22. Planeou o que iria comer durante o seu dia (ex., levou comida de casa; preparou a lista de compras; andou com snacks saudáveis)?

23. Planeou a atividade física que iria fazer durante o seu dia (ex., incorporou atividade física no trajeto para o trabalho; explorou tipos de atividade física que gostava; criou um plano de exercícios físicos)?

24. Permitiu a si própria consumir determinados alimentos/bebidas, sem limite de quantidades (ex., comeu/bebeu aquilo que quis, depois de um período de restrição; teve um dia/refeição de “asneira” ou fora do plano alimentar usual)?

25. Permitiu a si própria fazer atividade física mais leve (ou não fazer qualquer atividade física num determinado dia), depois de estar alguns dias a exercitar de forma regular?

26. Evitou comprar ou comer determinados alimentos (ex., evitou comer comida de que gosta muito)?

27. Evitou comer a determinadas horas do dia (ex., saltou a última refeição do dia ou comeu em menor quantidade)?

28. Evitou saídas com amigos/colegas ou determinados locais (ex., restaurantes), de forma a evitar comer certos alimentos

29. Estabeleceu regras para regular aquilo que comeu (ex., comeu devagar; levantou-se da mesa assim que acabou de comer; não comeu a comida que estava no prato até ao fim)?

30. Maximizou o potencial da atividade física, quando estava ativa (ex., percorreu o caminho mais longo sempre que ia a pé para o trabalho; andou com passo mais acelerado, em vez de andar mais lentamente)?

31. Limitou a sua alimentação, de forma rígida (ex., não ingeriu vários alimentos, com o objetivo de controlar ou perder peso)?

32. Limitou a atividade física que fez, de forma rígida (ex., fez constantemente apenas um tipo de atividade física, por acreditar que era a melhor para gerir o seu peso)?

33. Recompensou-se a si própria quando atingiu as metas a que se tinha proposto (ex., pôs dinheiro de parte para recompensar-se, depois de conseguir alcançar as suas metas)?

34. Estabeleceu horários para comer refeições ou comprar comida (ex., comeu em horários específicos, mesmo que não tivesse fome; comeu antes de ir comprar comida, para não ir com fome)?

35. Estabeleceu horários para fazer atividade física (ex., desistiu de outra coisa para ter tempo para fazer atividade física; fez atividade física num horário em que havia menos pessoas)?

36. Deitou-se, regularmente, à mesma hora?

37. Mediu ou pesou aquilo que comeu (ex., anotou as calorias, pesou os alimentos)?

38. Mediu a quantidade de atividade física que fez (ex., anotou o tempo despendido)?

39. Mediu o seu peso ou a sua forma corporal (ex., avaliou o quão apertada/larga estava a roupa, mediu a cintura com fita métrica)?

40. Limitou as quantidades disponíveis de comida, para controlar melhor aquilo que comeu (ex., usou pratos mais pequenos à refeição; comprou comida em menor quantidade; não teve em casa comida/bebidas não saudáveis)?

41. Deu dicas a si própria para fazer ou aumentar a prática de atividade física (ex., colocou um alarme a cada duas horas para se levantar e andar um pouco)?

42. Fez dieta juntamente com um amigo ou familiar?

43. Fez atividade física juntamente com um amigo ou familiar?

44. Pediu às pessoas à sua volta para não lhe oferecerem alimentos que estava a evitar comer?

45. Participou em grupos que fazem atividade física?

46. Procurou apoio ou partilhou com os outros (ex., família, colegas) os seus planos ou metas para gerir o seu peso?

47. Procurou ajuda profissional para gerir o seu peso (ex., médicos, nutricionistas, psicólogos, etc.)?
